# A comparative analysis of the effects of sevoflurane and propofol on cerebral oxygenation during steep Trendelenburg position and pneumoperitoneum for robotic-assisted laparoscopic prostatectomy

**DOI:** 10.1007/s00540-016-2241-y

**Published:** 2016-08-26

**Authors:** Aya Doe, Motoi Kumagai, Yuichiro Tamura, Akira Sakai, Kenji Suzuki

**Affiliations:** Department of Anesthesiology, School of Medicine, Iwate Medical University, 19-1 Uchimaru, Morioka, 020-0023 Japan

**Keywords:** Robotic-assisted laparoscopic prostatectomy (RALP), Steep Trendelenburg position, Cerebral oxygenation, Sevoflurane, Propofol

## Abstract

**Purpose:**

Steep Trendelenburg position and pneumoperitoneum during robotic-assisted laparoscopic prostatectomy (RALP) increase intracranial pressure (ICP) and may alter cerebral blood flow (CBF) and oxygenation. Volatile anesthetics and propofol have different effects on ICP, CBF, and cerebral metabolic rate and may have different impact on cerebral oxygenation during RALP. In this study, we measured jugular venous bulb oxygenation (SjO_2_) and regional oxygen saturation (SctO_2_) in patients undergoing RALP to evaluate cerebral oxygenation and compared the effects of sevoflurane and propofol. We also verified whether SctO_2_ may be an alternative to SjO_2_.

**Methods:**

Fifty patients scheduled for RALP were randomly assigned to undergo sevoflurane (group S) or propofol (group P) anesthesia. SjO_2_, SctO_2_, mean arterial pressure (MAP), heart rate (HR), cardiac index (CI), central venous pressure (CVP), partial pressures of arterial oxygen (PaO_2_) and carbon dioxide (PaCO_2_), hemoglobin concentration (Hb), Bispectral Index (BIS) and nasopharyngeal temperature (BT) were recorded 5 min before surgery commencement, 5 min after pneumoperitoneum, 5, 30, 60, 90, and 120 min after pneumoperitoneum in a Trendelenburg position, and after desufflation in a supine position.

**Results:**

SjO_2_ was significantly higher in group S than in group P at all measurement points [group S vs. group P: 77 % (11) vs. 65 % (13), mean of all measurement points (1SD); *p* < 0.01]. Linear regression analysis (*β* = 0.106; *r* ^2^ = 0.065; *p* = 0.004) shows a weak relationship between SjO_2_ and SctO_2_.

**Conclusions:**

Sevoflurane maintains higher SjO_2_ levels than propofol during RALP. SctO_2_ does not accurately reflect SjO_2_.

## Introduction

Robotic-assisted laparoscopic prostatectomy (RALP) requires a steep Trendelenburg position and pneumoperitoneum, both of which can increase intracranial pressure (ICP) [1, 2]. Increased ICP, in turn, may decrease cerebral perfusion pressure and cerebral oxygenation [3, 4].

Although sevoflurane and propofol are commonly used anesthetics, they have different effects on cerebral blood flow (CBF) and the cerebral metabolic rate for oxygen (CMRO_2_). Volatile anesthetics primarily increase CBF via local vasodilatation and reduce CMRO_2_. At low concentrations, where the effect of CMRO_2_ is dominant, these anesthetics constrict cerebral vessels by suppressing CMRO_2_ and prevent an increase in the CBF and CBF/CMRO_2_ ratio. At the medium-to-high concentrations commonly used in clinical settings, the direct vasodilatory effect becomes dominant, causing CBF and the CBF/CMRO_2_ ratio to increase. With sevoflurane, this effect is accompanied by an increase in cerebral blood volume (CBV) and an elevated ICP [5]. In RALP, where ICP is already increased, it is conceivable that the CBV increase caused by volatile anesthetics could further increase ICP and in turn lower the CBF/CMRO_2_ ratio and cerebral oxygenation. Supporting this hypothesis, one study showed cerebral oxygen desaturation in a steep Trendelenburg position and pneumoperitoneum with isoflurane anesthesia [4]. Propofol reduces CBF by cerebral vasoconstriction and suppresses CMRO_2_. The relationship between the vasoconstriction and CMRO_2_ suppression effects of propofol is unclear; some studies have reported approximate equivalency [6], and others have reported that vasoconstriction is slightly dominant [7]. Propofol at least does not increase the CBF/CMRO_2_ ratio. Volatile anesthetics and propofol each have the potential to decrease cerebral oxygenation during RALP.

Patients undergoing RALP are typically elderly and often have cerebrovascular disease that is associated with impaired cerebral oxygenation. These are not only associated with cerebral vasoconstriction or vasospasm during surgery but also with postoperative cognitive decline (POCD) [8]. Some studies suggest that the choice of anesthetic affects cerebral oxygenation and POCD [9, 10]. Thus, it is important to monitor cerebral oxygenation and choose anesthetics that best preserve cerebral oxygenation.

Jugular venous bulb oxygen saturation (SjO_2_) accurately reflects the CBF/CMRO_2_ ratio but is invasive and complex [11]. By contrast, near-infrared spectroscopy (NIRS) can non-invasively assess SctO_2_, which can reflect the CBF/CMRO_2_ ratio [12]. However, it has also been suggested that SctO_2_ and SjO_2_ are poorly correlated [12, 13].

To date, the difference in SjO_2_ between common anesthetic agents has not been investigated during RALP. In this study, we compared the effects of sevoflurane and propofol on SjO_2_, and verified whether SctO_2_ could be used as an alternative to SjO_2_ during RALP.

## Methods

The institutional ethics committee of Iwate Medical University Hospital, Japan, approved this study, and it has been registered in the UMIN Clinical Trials Registry (UMIN000016685). After obtaining written informed consent, we enrolled adult male patients who were undergoing RALP with the requirement that they were American Society of Anesthesiologists physical status class 1 or 2. Patients with known histories of cerebral ischemia or hemorrhage were excluded. Participants were randomized by computer generation into groups that received either sevoflurane (group S) or propofol (group P).

Anesthesia was induced in group S by a bolus injection of thiopental (5 mg/kg) and in group P by an effect-site target-controlled infusion (TCI) of propofol of 5 µg/ml; remifentanil (0.2–0.4 µg/kg/min) was used in both groups. After administering rocuronium (0.8 mg/kg), the trachea was intubated and the lungs were mechanically ventilated in volume control mode (tidal volume 8–10 ml/kg) with an oxygen fraction (FiO_2_) of 0.45. The respiratory rate was adjusted to maintain an end-tidal carbon dioxide tension (ETCO_2_) of 35–40 mmHg. In both groups, the concentrations of sedative drugs were adjusted to achieve a Bispectral Index (BIS) of 40–60. Sevoflurane was initiated at concentrations of 2.0 % and adjusted above 1.7 % (one minimum alveolar concentration; MAC), and propofol was maintained by TCI. Also, remifentanil was adjusted to maintain a mean arterial pressure (MAP) within 20 % of the preinduction value in both groups. When the MAP decreased to 80 % of the preinduction value, it was treated with an 8-mg bolus of ephedrine. Patients not anesthetized under these conditions were excluded from the final analysis.

Routine monitoring including electrocardiography, pulse oximetry, and non-invasive automated blood pressure measurement, started on arrival in the operating room. After the induction of anesthesia, a radial artery catheter was inserted for direct arterial blood pressure measurement and blood sampling. The arterial line was also connected to a FloTrac sensor and a third-generation Vigileo monitoring system (Edwards Lifesciences, Irvine, CA, USA) to monitor the cardiac index (CI) data. The ETCO_2_ and end-tidal concentration of sevoflurane were measured using a Vamos anesthetic gas monitor (Dräger Japan, Tokyo, Japan). Central venous pressure (CVP) was measured by a single-lumen central venous catheter that was inserted into the right internal jugular vein. A BIS electrode was applied to the forehead and was measured continuously using an Aspect BIS A2000 monitor v3.31 (Aspect Medical Systems, Natick, MA, USA). Nasopharyngeal temperature (BT) was monitored continuously.

For SctO_2_ measurement, the cerebral oximeter probes were placed bilaterally ≥1 cm above each eyebrow. SctO_2_ was monitored with an INVOS5100B cerebral oximeter (Somanetics, Troy, MI, USA), and the values from each side were averaged. For the continuous monitoring of SjO_2_, a Pre-Sep Oximetry Catheter (Edwards Lifesciences, Irvine, CA, USA) was connected to the Vigileo system and placed in the left jugular venous bulb. The catheter position was verified radiographically.

We recorded the SjO_2_, SctO_2_, MAP, heart rate (HR), CI, CVP, PaO_2_, PaCO_2_, hemoglobin concentration (Hb), BIS, and BT at the following points: 5 min before surgery (T0); 5 min after inducing a 15 mmHg pneumoperitoneum (T1); 5, 30, 60, 90, and 120 min after inducing the pneumoperitoneum in a 30° Trendelenburg position (T2, T3, T4, T5, and T6, respectively); and after desufflation in a supine position (T7).

Based on a pilot study, the sample size was calculated to detect a 9 % difference in the mean SjO_2_ value at T7 using G power: 68 % (±9 %) for group P compared with 77 % (±11 %) for group S. The power analysis indicated that we needed a minimum sample size of 17 patients per group to detect an effect size of 0.89 using independent Student’s* t* tests with an α of 0.05 and a power of 0.80. Thus, we aimed to include 25 patients per each group to allow for a 30 % drop-out rate. The Shapiro–Wilk test was used to test the normality of the data. Demographic variables, duration of surgery and anesthesia, ephedrine and remifentanil doses, blood loss, urine output, and intravenous fluid volume between groups were compared using unpaired Student’s *t* tests. A two-way repeated-measures analysis of variance (ANOVA) with post hoc unpaired *t* test and Bonferroni correction was used to compare SjO_2_, SctO_2_, MAP, HR, CI, CVP, PaO_2_, PaCO_2_, Hb, BT, and BIS between the groups. A one-way repeated-measures ANOVA with Bonferroni post hoc tests was used to analyze these variables across time within the groups. All statistical tests were two-tailed. The correlation between SjO_2_ and SctO_2_ was evaluated by simple linear regression. Significance was determined at *p* < 0.05, and we used XLSTAT 2015 for Windows (Addinsoft, New York, USA) for all analyses. Data are expressed as mean (standard deviation).

## Results

Of the 50 patients who were scheduled to enroll in the study, seven (three in group S and four in group P) were excluded. One patient in group P was found to have a history of cerebral infarction after consenting to the study and six withdrew their consent before the operation. In group P, an additional two patients were excluded, one because nicardipine was administered to treat intraoperative hypertension, and another because the FiO_2_ needed to be increased beyond the protocol limit to maintain the SaO_2_ >94 %. All of the measured values were normally distributed. There were no major complications on normal postoperative rounds in either group. As shown in Table 1, there were no significant differences in the demographic or anesthetic data between the groups.

**Table 1:** Demographic and anesthetic data

	Group S (*n* = 22)	Group P (*n* = 19)	*p* value
Age (years)	67 (4)	66 (4)	0.21
Weight (kg)	67 (8)	68 (6)	0.65
Height (cm)	165 (6)	166 (5)	0.22
Anesthetic time (min)	284 (39)	282 (37)	0.69
Operation time (min)	201 (40)	201 (32)	0.73
Fluid administered (ml)	1132 (362)	1103 (287)	0.78
Blood loss (ml)	94 (119)	108 (81)	0.65
Urine output (ml)	163 (124)	121 (82)	0.15
Ephedrine administered (mg)	47 (20)	40 (16)	0.11
Total dose of remifentanil administered (mg)	4.2 (1.2)	4.7 (0.8)	0.19

Data are presented as the mean (SD). There were no statistically significant differences between the two groups

**Fig. 1:**
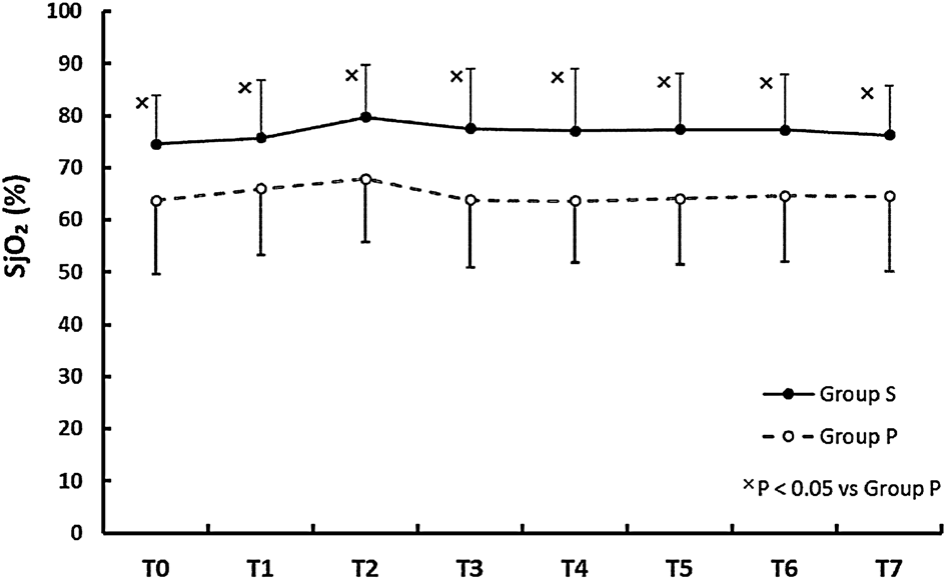
The time courses of jugular venous oxygen saturations (SjO_2_) in groups that received sevoflurane (*filled circle*) and propofol (*opened circle*). Values are expressed as mean (SD). *Multi symbol p* < 0.05 vs. group P

**Fig. 2:**
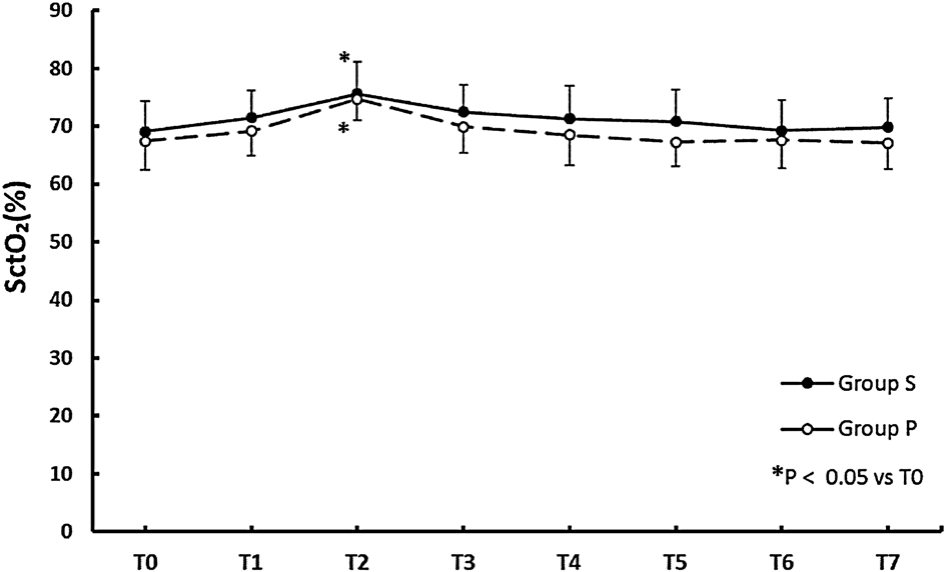
The time courses for regional oxygen saturations (SctO_2_) in groups receiving sevoflurane (*filled circle*) and propofol (*opened circle*). Values are expressed as mean (SD). *p < 0.05 vs. T0

Figure 1 shows that the SjO_2_ was significantly higher in group S at all measurement points (*p* < 0.05) with no significant changes throughout the procedure when compared with T0. By contrast, Fig. 2 shows that there was no significant difference in the SctO_2_ values between the groups at each point, but that SctO_2_ was significantly higher at T2 than at T0 within each group.

**Fig. 3:**
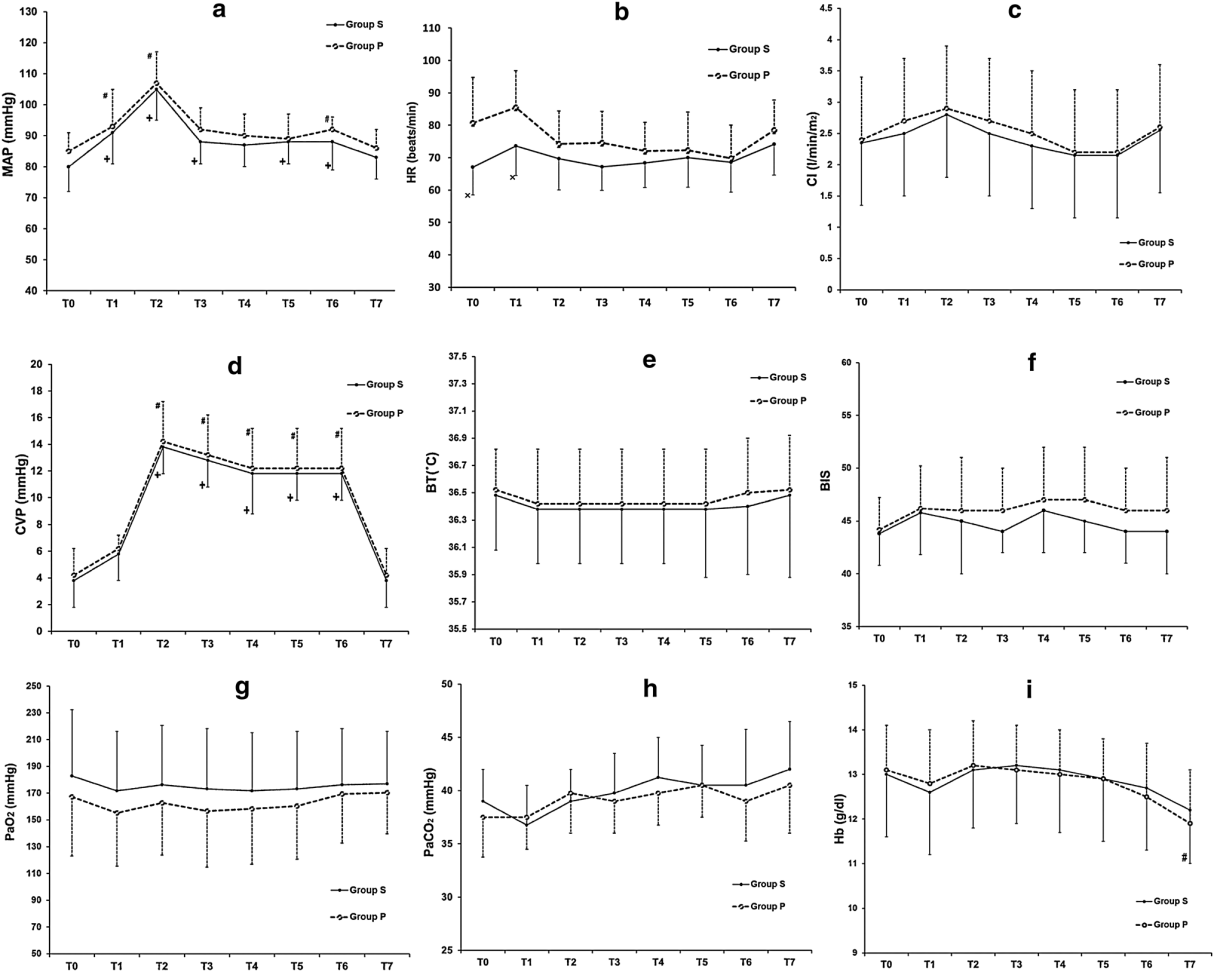
Changes in **a** mean arterial pressure (MAP), **b** heart rate (HR), **c** cardiac index (CI), **d** central venous pressure (CVP), **e** nasopharyngeal temperature (BT), **f** Bispectral Index (BIS), **g** partial pressures of arterial oxygen (PaO_2_), **h** partial pressures of carbon dioxide (PaCO_2_), and **i** hemoglobin concentration (Hb). Measurements were performed: 5 min before the commencement of surgery (T0); 5 min after a 15 mmHg pneumoperitoneum (T1); 5, 30, 60, 90 and 120 min after the Trendelenburg position (T2, T3, T4, T5, and T6, respectively); and after exsufflation in the supine position (T7). *Plus symbol p* < 0.05 vs. T0 in group S. *Hash symbol p* < 0.05 vs. T0 in group P. *Multi symbol p* < 0.05 vs. group P

Figure 3 shows that there were no differences across the measured time points between the groups in most of the key parameters that could affect SjO_2_ and SctO_2_. However, HR in group S was lower than that in group P at T0 and T1. Compared with T0, the MAP was significantly higher at T1–T6 in group S, and at T1, T2, and T6 in group P. In both groups, CVP was significantly higher during pneumoperitoneum in the Trendelenburg position (T2–T6) compared with T0. In group P, Hb was significantly lower at T7 than at T0. Compared with T0, there were no differences within each group for PaO_2_, PaCO_2_, CI, Hb, BT, and BIS at each time point.

**Fig. 4:**
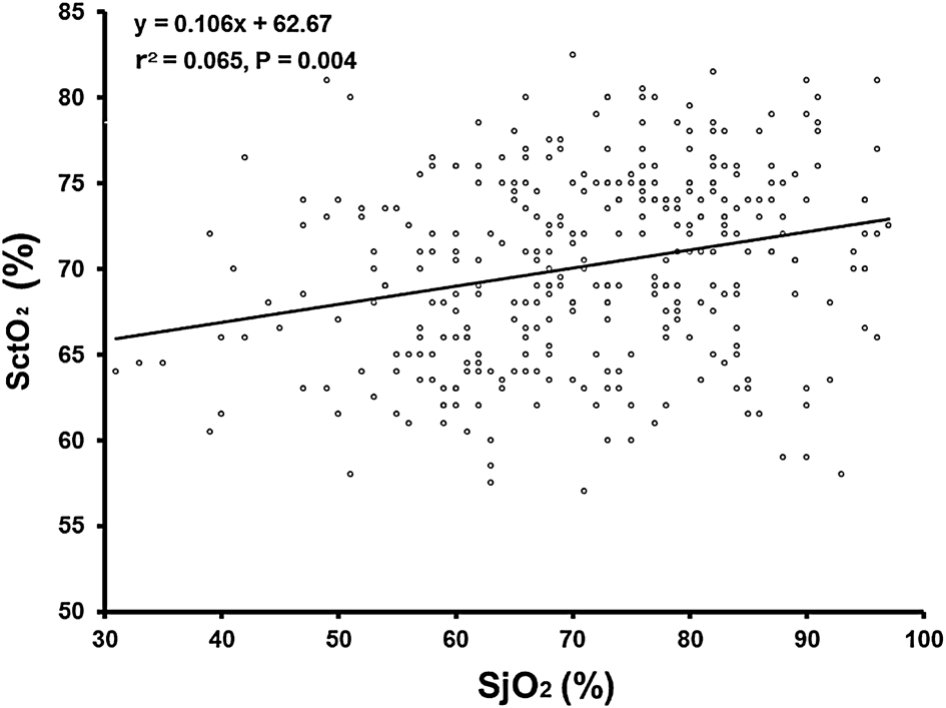
Linear regression showing a weak relationship between regional oxygen saturation (SctO_2_) and jugular venous oxygen saturation (SjO_2_). Data are for 328 measurement pairs from 41 patients

Figure 4 shows the linear regression analysis for SjO_2_ and SctO_2_ during anesthesia, with evidence of only a weak relationship (*β* = 0.106; *r* ^2^ = 0.065; *p* = 0.004).

## Discussion

The present study is the first to compare cerebral oxygenation by SjO_2_ between anesthetic regimens during RALP and showed that sevoflurane had significantly higher levels of SjO_2_ than propofol at all measurement points. We also observed no intergroup differences in MAP, CI, CVP, BT, BIS, PaO_2_, PaCO_2_, Hb at any time point and in the total ephedrine and remifentanil dose, even though HR was lower in group S at T0 and T1. The difference in HR may be explained by the higher doses of ephedrine administered in group P to treat hypotension during this period. Although not statistically significant, the PaO_2_ of group S was higher by about 15 mmHg at all measurement points, but this is likely too small to affect SjO_2_ physiologically and also from a literature review [14]. BT and BIS have been strongly related to cerebral metabolism [15, 16], but there were no significant differences in these values between the groups suggesting that there was no difference in CMRO_2_. Because we used sevoflurane at a mid-range concentration (above 1 MAC), we presumed that the cerebrovascular dilation effect was dominant in group S and that the intergroup differences in SjO_2_ were due to the differences in CBF.

Cerebral oxygen desaturation during surgery can predict early POCD and has been associated with delirium and prolonged hospitalization among the elderly [17, 18]. Current study might show that sevoflurane is better than propofol for RALP, but the result of our study does not directly indicate that sevoflurane is a superior choice for RALP. Cerebrovascular dilation effect of sevoflurane also implies the cerebrovascular dysregulation, which can be amplified during RALP [19] and could result in hyperemia and cerebral edema. These aspects must be clarified if we are to demonstrate the superiority of sevoflurane over propofol in a clinical setting because they might occur prior to a decrease in SjO_2_ or cerebral perfusion pressure. Although we performed no cognitive tests, our study might suggest the appropriateness of sevoflurane over propofol for patients with lower baseline values for SjO_2_ due to cerebrovascular stenosis during pneumoperitoneum and a steep Trendelenburg position. A study comparing sevoflurane and propofol anesthesia among patients with baseline SjO_2_ values <50 % showed no differences in postoperative cognitive function [20], but the study did not show the SjO_2_ difference between the groups and did not control for BP, CI, or PaCO_2_, so we cannot assess the true relationship between the anesthetics, SjO_2_, and postoperative cognitive function. Several studies have shown the differences in cerebral oxygenation depending on the anesthetic used in a variety of clinical settings, but only a few have done so for pneumoperitoneum in the Trendelenburg position. One study tested whether SctO_2_ differed between propofol and sevoflurane anesthesia during laparoscopic surgery in a 20° Trendelenburg position [21], and concluded that propofol was associated with significantly lower SctO_2_ than sevoflurane. In situations of raised ICP (e.g., brain tumors), research with volatile anesthetics have shown that they may maintain a significantly higher SjO_2_ compared with propofol [22]. Further, in the supine position for normal surgery [23], in the sitting position for arthroscopy [13], and during one-lung ventilation for lung surgery [24], volatile anesthetics can maintain higher cerebral oxygenation than propofol. One pilot study has reported that, compared with sevoflurane, propofol increased cerebral SctO_2_ only when the stimulus of surgery was intense [25]. Although the authors speculated that an increased propofol dose due to the stimulation caused the CMRO_2_ to decrease, the mechanism remains unclear because the concentrations of sevoflurane were not disclosed. Most of the studies discussed above, including the current study, indicate that volatile anesthetics cause higher cerebral oxygenation levels with no differences in neurological complications or POCD when compared with propofol anesthesia. We also showed that SjO_2_ was well preserved in both groups (>50 %; Fig. 1). Although volatile anesthetics and propofol presumably have the potential to decrease cerebral oxygenation during RALP, we showed that neither did so. Considering the lack of significant changes in SjO_2_ compared with the baseline (T0), it is conceivable that pneumoperitoneum and a 30° Trendelenburg position may not affect cerebral oxygenation within 2 h. This may be because the predicted raise in ICP was within the physiological compensation, such as cerebrospinal fluid (CSF) and blood moving into the spinal canal and extracranial vasculature not to suppress cerebral oxygenation, and also may be due to the effect of cerebrovascular autoregulation although that might be partly impaired in group S.

We also found only a weak relationship between SjO_2_ and SctO_2_. There are several factors that could contribute to the inconsistency between the two values. First, SjO_2_ is an index of oxygenation for the entire brain, whereas SctO_2_ is an index of localized oxygenation in the frontal lobe [9–11, 26]. Second, NIRS measures blood oxygenation in arteries, veins, and capillaries combined; and is affected by extracerebral blood flow, cerebrospinal fluid, and systemic blood pressure. By contrast, SjO_2_ measures only the intracerebral mixed venous saturation [27, 28]. Interestingly, in this study, SctO_2_ showed similar changes to BP across time, whereas SjO_2_ did not. Third, NIRS calculates SctO_2_ based on a fixed artery-to-vein ratio, regardless of changes induced by the Trendelenburg position and anesthetic agents [9, 28].

The present study has several limitations. First, we did not routinely perform cognitive tests. Thus, it remains unclear whether postoperative cognitive outcomes correlate with differences in cerebral oxygenation by different anesthetic agents; further research is needed. Second, we measured SjO_2_ unilaterally, and although no research has shown a clear difference between left and right SjO_2_, this may have affected the results. Third, we did not include patients with cerebrovascular disease and complications, so we cannot state how the SjO_2_ or SctO_2_ would have responded to RALP in patients with a cerebral pathology. Fourth, the values for ICP, CBF, and CMRO_2_ were not measured directly, which should be rectified in future research. Fifth, we limited the time spent with a pneumoperitoneum in the Trendelenburg position to 120 min, yet there have been reports of neurological complications due to brain edema when this procedure exceeds 8 h [29]. Actually, cerebrovascular autoregulation gradually changes with prolonged pneumoperitoneum in the Trendelenburg position; one study showed that it is decreased from 170 min [19]. Considering the inhibitory effect of volatile anesthetics on cerebrovascular autoregulation, it may be worth comparing the effects of longer surgery and steeper Trendelenburg positions in the future.

We conclude that sevoflurane allows for a higher SjO_2_ than propofol during RALP. We also conclude that SctO_2_ does not accurately reflect SjO_2_ during this procedure. Ultimately, further research is needed to determine the effect of anesthetics on hyperemia and cerebral edema during longer pneumoperitoneums and in steeper Trendelenburg positions, as well as on the potential correlation between postoperative cognitive outcomes and differences in cerebral oxygenation between anesthetic agents.
